# Exploration of the association between estimated glucose disposal rate and osteoarthritis in middle-aged and older adults: An analysis of NHANES data from 2011 to 2018

**DOI:** 10.1515/med-2024-1120

**Published:** 2025-02-04

**Authors:** XiaoPeng Gu, SongOu Zhang, WeiHu Ma

**Affiliations:** Department of Clinical Medicine, Health Science Center, Ningbo University, Ningbo City, Zhejiang, P. R. China; Department of Orthopedics, NingBo No. 6 Hospital, Ningbo City, Zhejiang, P. R. China; Department of Orthopedics, Zhoushan Guhechuan Hospital, Zhoushan City, Zhejiang, P. R. China; Department of Orthopedics, Zhoushan Institute of Orthopedics and Traumatology, Zhoushan City, Zhejiang, P. R. China

**Keywords:** NHANES, eGDR, osteoarthritis, diabetes mellitus, insulin resistance

## Abstract

**Background:**

It is unclear how the estimated glucose disposal rate (eGDR) index relates to osteoarthritis (OA). The goal of this research is to explore the possible link between the eGDR index and the likelihood of OA development.

**Methods:**

The study encompassed 9,051 individuals from the National Health and Nutrition Examination Survey (2011–2018). Participants were divided into quartiles according to their eGDR, calculated with the equation: eGDR (mg/kg/min) = 21.158 − (0.09 × waist circumference) − (3.407 × hypertension) − (0.551 × glycosylated hemoglobin). We assessed the independent correlation between the eGDR metric and the incidence of OA through weighted multivariate regression, stratified analysis, and threshold effect evaluation.

**Results:**

The study encompassed 9,051 participants, who had an average eGDR of 7.09. Participants with OA had lower eGDR levels compared to those without OA (6.27 ± 0.09 vs 7.31 ± 0.06, *P* < 0.001). The odds ratios (ORs) for OA associated with the eGDR index in the logistic regression models were 0.87 (95% confidence interval [CI]: 0.84, 0.89) in the unadjusted model I and 0.87 (95% CI: 0.84, 0.91) in model II (adjusted for all covariates). Higher eGDR index was associated with a reduced risk of OA when compared to the lowest quartile (Q1). A restricted cubic spline analysis indicated a linear negative relationship between eGDR and OA risk.

**Conclusion:**

An increased eGDR index is inversely related to the risk of OA. The eGDR may serve as a valuable biomarker for the detection of OA and offers a new perspective for the assessment and management of the condition.

## 1 Introduction

Osteoarthritis (OA) is a chronic joint disorder characterized by cartilage degradation, bone proliferation adjacent to the joints, and inflammation of the synovial membrane. It ranks among the primary sources of discomfort and impairment among the elderly population. OA represents a significant global public health issue, especially affecting middle-aged and older individuals aged 50 and over. According to statistics, the incidence of OA ranges from 9.29 to 35.1% [1,2,3], and this figure is continuously rising with the aging of the population. The pathogenesis of OA involves multiple aspects, including the degenerative changes of articular cartilage, joint inflammation, and abnormal bone remodeling, leading to symptoms such as joint pain, swelling, and limited mobility. In severe cases, it can result in joint deformity and loss of function. Currently, the treatment for OA primarily includes pharmacological therapy (such as non-steroidal anti-inflammatory drugs and analgesics), physical therapy, and surgical intervention, but no definitive treatment exists that can reverse joint damage. OA not only inflicts physical suffering on patients but also imposes a significant economic burden on society. Medical expenses, loss of labor force, and decreased productivity are socioeconomic issues associated with OA. Regarding the etiology of OA, in addition to age and genetic factors, obesity, excessive joint use, and metabolic abnormalities are also considered important risk factors [4,5,6,7,8].

Diabetes mellitus, a common metabolic disorder, is characterized by persistently elevated blood glucose levels, which are typically the result of insufficient insulin production or inadequate response to insulin by the body [9]. There are two main forms of diabetes mellitus: type 1 and type 2 diabetes. The relationship between diabetes and OA is increasingly being recognized. Obesity is a shared risk factor for both diseases, potentially increasing the risk of developing either condition. However, obesity alone does not solely account for the occurrence of OA. Studies have indicated that even after accounting for obesity, diabetes may still increase the risk of OA [10]. Diabetes mellitus may lead to an elevation in systemic inflammation levels, which could potentially accelerate joint degeneration. The metabolic disturbances associated with diabetes may also affect joint health. Insulin resistance (IR), a precursor to diabetes, is considered a potential risk factor for the onset of OA [11,12,13]. Traditional methods for detecting IR, such as the euglycemic hyperinsulinemic clamp, are accurate but are limited in clinical application due to their high cost and invasiveness [14]. In contrast, the estimated glucose disposal rate (eGDR) is a convenient and non-invasive assessment method that calculates IR based on clinical features such as waist circumference (WC), hypertension, and glycosylated hemoglobin (HbA1c). eGDR has a strong correlation with IR [15]. A lower eGDR value indicates more severe IR [16].

Although eGDR has advantages in assessing IR, its association with OA remains unclear. Recently, a growing body of research has indicated a possible connection between IR and OA. For example, IR might result in metabolic disruptions in articular chondrocytes, which could aggravate joint inflammation and the degeneration of cartilage [17]. Drawing on the findings of these investigations, we propose that eGDR could function as an indicator for OA in middle-aged and older individuals.

The NHANES is a nationwide survey conducted by the National Center for Health Statistics within the Centers for Disease Control and Prevention in the United States, with the objective of assessing the health and nutritional status of the population. Data collected via NHANES are widely utilized in scholarly research, policy formulation, and public health monitoring [18,19,20]. This study leverages data from the US NHANES from 2011 to 2018 to conduct an in-depth analysis of the relationship between the eGDR index and OA in middle-aged and elderly populations. Our findings indicate that the incidence of OA among middle-aged and elderly individuals with lower eGDR values is significantly higher than that among those with higher eGDR values. The results of this study provide new insights into the role of IR in the pathogenesis of OA and offer a theoretical basis for the clinical practice of preventing and managing OA by improving insulin sensitivity. Future research can further explore the role of eGDR in predicting OA risk and treatment response, with the aim of providing more evidence for precise treatment of OA.

## 2 Methods

### 2.1 Study population

**Figure 1:**
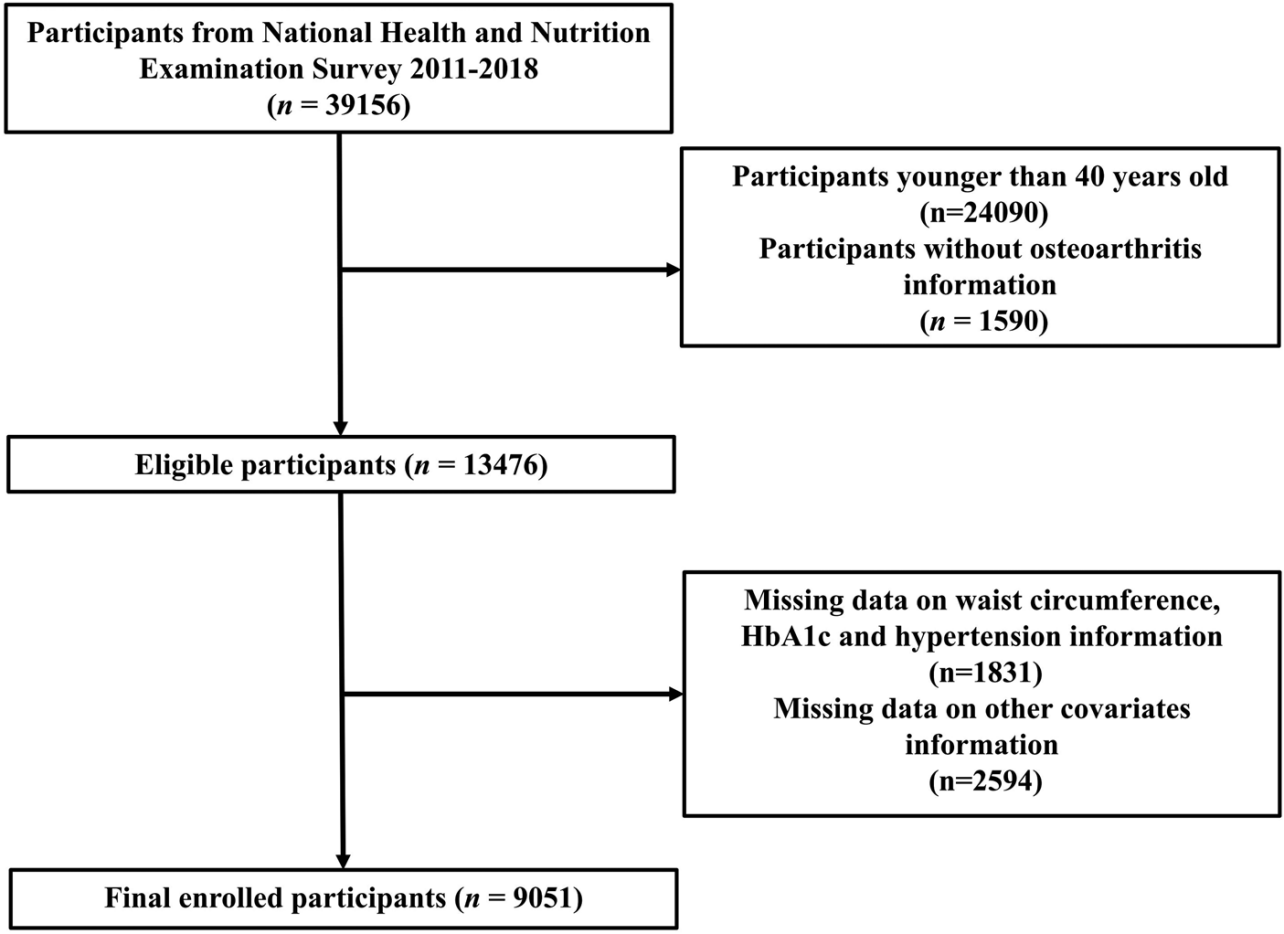
An elaborate diagram of the participant enrollment process.

This study initially included cross-sectional data from 39,156 participants across four consecutive NHANES cycles (2011–2018). The exclusion criteria were as follows: (1) participants under 40 years of age (*n* = 24,090); (2) participants lacking information on OA (*n* = 1,590); (3) participants with missing data on WC, HbA1c, and hypertension, which are necessary for the calculation of eGDR (*n* = 1,831); and (4) participants with missing information on other covariates (*n* = 2,594). After a manual review of the data, a total of 9,051 subjects were chosen for subsequent analysis. Figure 1 provides a detailed depiction of the participant enrollment flow for the study.

### 2.2 Assessment of eGDR

eGDR stands for the estimated glucose disposal rate, a metric that represents the milligrams of glucose disposed per kilogram of body weight per minute. The calculation of eGDR is based on the following formula, which incorporates WC (in centimeters), the presence of hypertension (yes = 1/no = 0), and the percentage of HbA1c. The calculation of eGDR is based on the following formula: eGDR (mg/kg/min) = 21.158 − (0.09 × WC) − (3.407 × hypertension) − (0.551 × HbA1c).

### 2.3 Assessment of OA

NHANES’s “Medical Conditions” section provided diagnostic data for OA. Initially, participants were inquired whether a physician had ever informed them of having OA. If they responded affirmatively, they were subsequently prompted to specify the type of OA diagnosed (according to the NHANES questionnaire data, OA is categorized into osteoarthritis, rheumatoid arthritis, and psoriatic arthritis, among others).

### 2.4 Covariates

In this study, we included several covariates that might affect the risk of OA. Ethnicity was categorized into five groups: White, Black, Hispanic, and other racial/ethnic groups. Education level was divided into less than high school and high school or above. The poverty income ratio (PIR) was used as an indicator of household income, which has been linked to human health in various studies. PIR was classified into three categories: <1.3%, 1.3–3.49%, and ≥3.5%. Participants were classified as drinkers if they reported having consumed a minimum of 12 drinks within the year prior to the survey. No matter whether they smoked at the time of the interview or not, smokers were classified as those who had smoked 100 or more cigarettes in their lifetime. Blood pressure measurements, including systolic blood pressure (SBP) and diastolic blood pressure (DBP), were taken by clinicians following standardized procedures, with three successive readings at 30 s intervals, and the average of these was employed to determine the blood pressure value. Laboratory tests were conducted following standardized procedures to determine serum triglyceride concentration, total cholesterol (TC), and HbA1c. To calculate the estimated glomerular filtration rate (eGFR), the NHANES researchers applied the Chronic Kidney Disease Epidemiology Collaboration (CKD-EPI) formula, which takes into account age, sex, race/ethnicity, and serum creatinine concentrations to cater to a varied demographic. Hypertension was identified based on either self-reported diagnoses by a physician or readings from a physical examination. Study participants were deemed hypertensive if they fulfilled one or more of the following criteria: (1) an average SBP of at least 140 mmHg; (2) an average DBP of at least 90 mmHg; (3) a self-reported diagnosis of hypertension; and (4) the current use of antihypertensive medications. Participants were considered to have diagnosed diabetes if a medical professional had identified the condition. Individuals who had not been previously diagnosed but exhibited HbA1c levels equal to or greater than 6.5% (47.5 mmol/mol), fasting plasma glucose levels of 126 mg/dL (7.0 mmol/L) or higher, or 2 h post-meal plasma glucose levels of 200 mg/dL (11.1 mmol/L) or higher following an oral glucose tolerance test were classified as having undiagnosed diabetes. Participants with either diagnosed or undiagnosed diabetes were collectively referred to as diabetic.

### 2.5 Statistical analysis

The method employed facilitated the precise computation of associated statistical data, accounting for the intricate sampling strategy inherent to the NHANES survey. Continuous variables are described by their mean and standard deviation, while nominal variables are depicted by frequency and percentage. Student’s *t*-tests and chi-square tests were applied to discern baseline differences in characteristics between participants with and without OA. eGDR values were divided into four quartiles, with the first quartile (Q1) serving as the reference category. The analysis comprised an unadjusted model and two adjusted models (model I and model II) to evaluate the relationship between eGDR and OA. Model I included adjustments for years, gender, and race, whereas model II accounted for educational level, poverty–income ratio, smoking habits, alcohol intake, body mass index (BMI), hemoglobin levels, TC, and the presence of diabetes and hypertension.


**Informed consent:** This is a study based on a public database. Our team did not need to obtain patient informed consent before conducting the study.
**Ethical approval:** This is a study based on a public database. Our team did not need to obtain additional ethics approval. We put the ethics approval of the NHANES database in the Supplementary Material.

## 3 Result

### 3.1 Descriptive statistics of clinical features in the study cohort

A total of 9,051 participants were included in the analysis, with a weighted average age of 57.35 ± 0.22 years. These individuals were divided into OA and non-OA categories, with the prevalence of OA across the entire participant pool standing at 72.81%. Compared to individuals without OA, those with OA were generally older and predominantly Caucasian and exhibited significant differences in BMI, hemoglobin, WC, HbA1c, eGDR, TC, eGFR, and smoking status (all *P*  <  0.05). Among those with OA, there was a higher proportion of Caucasians (83.70% versus 69.88% in non-OA individuals). They tended to be older (63.23 ± 0.31 years versus 55.77 ± 0.22 years), had higher BMI (31.10 ± 0.26 versus 29.07 ± 0.12), and had larger WCs (105.32 ± 0.54 cm versus 101.07 ± 0.28 cm). They also had a higher incidence of diabetes (24.84% versus 18.62%) and hypertension (63.84% versus 45.78%) and had lower hemoglobin levels (105.32 ± 0.54 g/L versus 101.07 ± 0.28 g/L). The detailed demographic and clinical characteristics of all participants are presented in Table 1. This table shows the demographic and clinical characteristics of the study population grouped by eGDR quartiles. In the OA group, eGDR values were notably lower compared to the entire study cohort. (6.27 ± 0.09 versus 7.31 ± 0.06) (Table 1).

**Table 1:** Clinical characteristics of the study population

Variables	Overall	Non-OA	OA	*P* value
Age (%)	57.35 ± 0.22	55.77 ± 0.22	63.23 ± 0.31	<0.001***
Race/ethnicity (%)				<0.001***
White	72.81 (64.65, 80.97)	69.88 (66.27, 73.49)	83.70 (81.31, 86.09)	
Black	8.95 (7.67, 10.24)	9.78 (7.95, 11.61)	5.89 (4.52, 7.26)	
Mexican	6.26 (4.85, 7.67)	7.12 (5.44, 8.79)	3.06 (2.01, 4.12)	
Others	11.98 (10.73, 13.23)	13.23 (11.51, 14.95)	7.34 (5.99, 8.69)	
Education levels, %				0.01*
Less than high school	12.72 (11.10, 14.35)	13.37 (11.56, 15.18)	10.32 ( 8.49, 12.15)	
High school or equivalent	22.18 (19.77, 24.59)	22.40 (20.67, 24.13)	21.37 (18.51, 24.22)	
College or above	65.10 (59.45, 70.75)	64.23 (61.50, 66.96)	68.32 (65.34, 71.29)	
PIR, %				0.40
≤1.30	16.43 (14.40, 18.45)	16.74 (14.65, 18.82)	15.28 (12.78, 17.78)	
1.31–3.49	34.31 (31.39, 37.24)	34.44 (32.35, 36.54)	33.84 (30.98, 36.71)	
≥3.50	49.26 (44.02, 54.50)	48.82 (45.60, 52.04)	50.88 (46.70, 55.06)	
BMI (kg/m^2^)	29.50 ± 0.12	29.07 ± 0.12	31.10 ± 0.26	<0.001***
Hemoglobin (g/dl)	14.19 ± 0.03	14.23 ± 0.04	14.02 ± 0.04	<0.001***
waist circumference (cm)	101.97 ± 0.28	101.07 ± 0.28	105.32 ± 0.54	<0.001***
HbA1c (%)	5.81 ± 0.02	5.80 ± 0.02	5.86 ± 0.03	0.05
eGDR	7.09 ± 0.05	7.31 ± 0.06	6.27 ± 0.09	<0.001***
TC (mmol/L)	5.15 ± 0.02	5.16 ± 0.02	5.11 ± 0.04	0.25
eGFR (mL/min/1.73 m^2^)	85.77 ± 0.35	87.24 ± 0.38	80.30 ± 0.60	<0.001***
Smoking (%)	45.78 (41.53, 50.02)	44.52 (42.62, 46.43)	50.43 (46.54, 54.33)	0.004**
Drinking (%)	76.64 (70.83, 82.45)	77.05 (75.18, 78.92)	75.13 (72.04, 78.21)	0.20
Hypertension (%)	49.61 (46.11, 53.11)	45.78 (43.84, 47.72)	63.84 (60.46, 67.22)	<0.001***
DM (%)	19.94 (18.24, 21.63)	18.62 (17.32, 19.91)	24.84 (21.94, 27.74)	<0.001***

Continuous data were presented as the mean ± SEM, and category data were presented as the proportion and 95% confidence interval. SEM, standard error of the mean; OA, osteoarthritis; PIR, poverty income ratio; BMI, body mass index; HbA1c, glycosylated hemoglobin; eGDR, estimated glucose disposal rate; TC, total cholesterol; eGFR, estimated glomerular filtration rate; DM, diabetes mellitus; ***P value <0.001, **P value <0.01, *P value <0.05.

### 3.2 Association between eGDR and the incidence of OA

**Figure 2:**
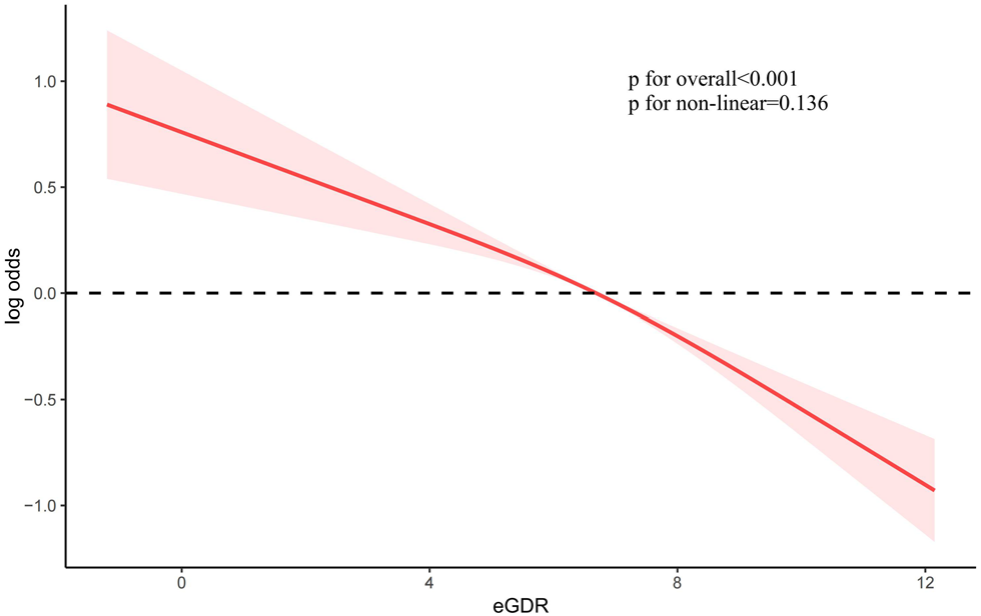
The relationship between eGDR and OA as depicted in a constrained cubic spline regression model.

**Figure 3:**
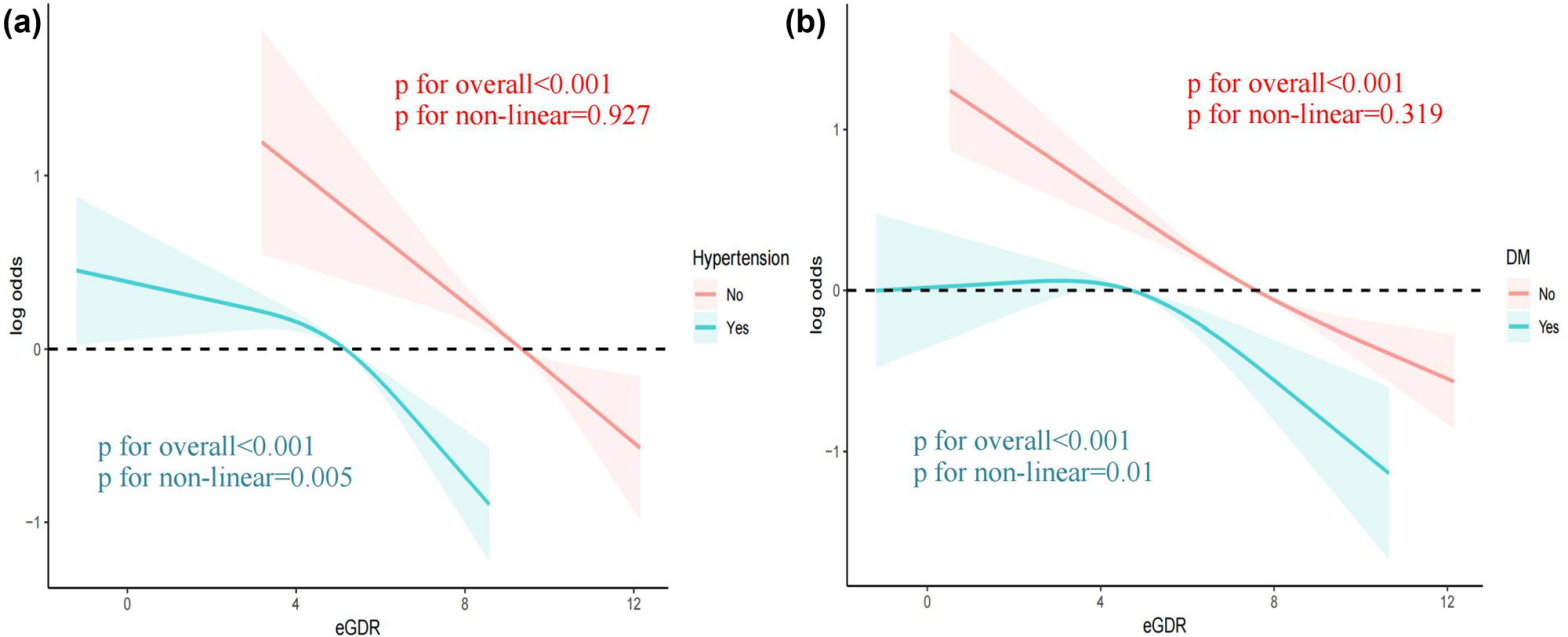
Restricted cubic spline analyses illustrating the relationship between eGDR and OA within subgroups, stratified by (a) hypertensive status and (b) diabetes mellitus.

To investigate the potential link between eGDR and the development of OA, we conducted a weighted multivariate analysis, adjusting for variables including age, sex, ethnicity, educational level, BMI, PIR, smoking habits, alcohol consumption, hypertension, and diabetes. A notable correlation between eGDR and the frequency of OA was detected, both in unadjusted and adjusted models for confounders, suggesting that an increased eGDR, when considered a continuous measure, is linked to a lower likelihood of developing OA. The odds ratio (OR) (95% confidence interval [CI]) for the relationship between eGDR and the incidence of OA was 0.87 [0.84–0.89] (*P* < 0.001) (refer to Table 2). Furthermore, by uniformly dividing all participants into quartiles based on eGDR, we observed that participants with higher eGDR quartiles had a significantly lower prevalence of OA (Table 2). Constrained cubic spline functions were employed to assess the association between eGDR and the occurrence of OA. The results indicate a linear inverse relationship between eGDR and the risk of OA (non-linear *P* = 0.136) (Figure 2). With increasing eGDR, the incidence of OA decreased more prominently. Constrained cubic spline techniques were utilized to investigate the correlation between eGDR and the frequency of OA among individuals with diabetes and hypertension. The results demonstrate a non-linear inverse relationship between eGDR and OA prevalence in these patient groups (diabetes: non-linear *P* = 0.005; hypertension: non-linear *P* = 0.01) (Figure 3). With increasing eGDR, the decrease in the incidence of OA was more pronounced.

**Table 2:** Weighted logistic regression analysis on the association between eGDR and OA

	Non-adjusted model		Model I		Model II	
	OR (95% CI)	*P* value	OR (95% CI)	*P* value	OR (95% CI)	*P* value
Continuous eGDR	0.87 (0.84, 0.89)	<0.001***	0.88 (0.85, 0.91)	<0.001***	0.87 (0.84, 0.91)	<0.001***
eGDR-Q1	Reference	—	Reference	—	Reference	—
eGDR-Q2	0.77 (0.62, 0.94)	0.01*	0.69 (0.55, 0.86)	0.001**	0.68 (0.53, 0.87)	0.003**
eGDR-Q3	0.51 (0.41, 0.63)	<0.001***	0.51 (0.40, 0.64)	<0.001***	0.50 (0.39, 0.64)	<0.001***
eGDR-Q4	0.34 (0.26, 0.44)	<0.001***	0.38 (0.29, 0.50)	<0.001***	0.37 (0.27, 0.51)	<0.001***

Data are presented as OR (95% CI). Model I adjusted for age, sex, and race/ethnicity. Model II adjusted for age, sex, race/ethnicity, education levels, poverty income ratio, smoking, drinking, DM, hemoglobin, TC, and eGFR. OA, Osteoarthritis; eGDR, estimated glucose disposal rate; TC, total cholesterol; eGFR, estimated glomerular filtration rate; ***P value < 0.001, **P value < 0.01, *P value < 0.05.

Results are expressed as ORs (95% CIs). Model I included adjustments for age, gender, and ethnicity. Model II was adjusted for age, gender, ethnicity, educational attainment, poverty–income ratio, tobacco use, alcohol consumption, diabetes mellitus, hemoglobin levels, total cholesterol, and estimated glomerular filtration rate. OA: osteoarthritis; eGDR: estimated glucose disposal rate; TC: total cholesterol; eGFR: estimated glomerular filtration rate; *** indicates *P*-value <0.001, ** indicates *P*-value <0.01, * indicates *P*-value <0.05.

### 3.3 Sensitivity analysis

To ascertain the consistency of our study’s outcomes, we performed a sensitivity analysis with unweighted logistic regression. This sensitivity analysis confirmed a statistically significant relationship between eGDR and the incidence of OA, with higher eGDR scores inversely related to the risk of OA. For each unit increment in eGDR, the OR for OA prevalence was 0.87 (95% CI: 0.85–0.88) (refer to Table 3). The unweighted logistic regression model applied in the sensitivity analysis underscored the reliability of our findings, suggesting that the correlation between eGDR and OA prevalence aligns with the initial analysis.

**Table 3:** Unweighted logistic regression analysis on the correlation between eGDR and OA in sensitivity analysis

	Non-adjusted model		Model I		Model II	
	OR (95% CI)	*P* value	OR (95% CI)	*P* value	OR (95% CI)	*P* value
Continuous eGDR	0.87 (0.85, 0.88)	<0.001***	0.88 (0.86, 0.90)	<0.001***	0.87 (0.85, 0.89)	<0.001**
eGDR-Q1	Reference	—	Reference	—	Reference	—
eGDR-Q2	0.75 (0.65, 0.86)	<0.001***	0.67 (0.57, 0.77)	<0.001***	0.65 (0.56, 0.76)	<0.001***
eGDR-Q3	0.50 (0.43, 0.58)	<0.001***	0.50 (0.43, 0.59)	<0.001***	0.49 (0.41, 0.57)	<0.001***
eGDR-Q4	0.35 (0.29, 0.41)	<0.001***	0.41 (0.34, 0.49)	<0.001***	0.39 (0.32, 0.47)	<0.001***

Data are presented as OR (95% CI). Model I adjusted for age, sex, and race/ethnicity. Model II adjusted for age, sex, race/ethnicity, education levels, poverty income ratio, smoking, drinking, DM, hemoglobin, TC, and eGFR. OA, osteoarthritis; eGDR, estimated glucose disposal rate; TC, total cholesterol; eGFR, estimated glomerular filtration rate; ***P value < 0.001, **P value < 0.01.

### 3.4 Subgroup analysis across different populations

Stratified analyses were performed to investigate the potential relationships between eGDR and the incidence of OA, classified according to age, sex, ethnicity, BMI, educational status, PIR, smoking habits, alcohol use, hypertension, and diabetes (refer to Table 4). eGDR might interact with the prevalence of OA across various age strata, among smokers, and in individuals with diabetes.

**Table 4:** Subgroup analysis for the association between the eGDR and OA

Variable name	Non-OA	OA	*P* value	*P* for interaction
Age				0.001
40–60 years	Ref	0.85 (0.82, 0.89)	<0.001	
>60 years	Ref	0.94 (0.90, 0.98)	0.01	
Sex				0.59
Male	Ref	0.84 (0.81, 0.88)	<0.001	
Female	Ref	0.85 (0.82, 0.88)	<0.001	
BMI				0.50
≤30	Ref	0.87 (0.83, 0.91)	<0.001	
>30	Ref	0.89 (0.85, 0.93)	<0.001	
Race				0.06
White	Ref	0.87 (0.84, 0.90)	<0.001	
Black	Ref	0.90 (0.84, 0.95)	<0.001	
Mexican American	Ref	0.83 (0.78, 0.89)	<0.001	
Others	Ref	0.81 (0.76, 0.86)	<0.001	
Education levels				0.23
Less than high school	Ref	0.83 (0.78, 0.88)	<0.001	
High school or equivalent	Ref	0.84 (0.77, 0.90)	<0.001	
College or above	Ref	0.88 (0.85, 0.90)	<0.001	
PIR				0.91
≤1.30	Ref	0.87 (0.82, 0.91)	<0.001	
1.31–3.49	Ref	0.86 (0.82, 0.89)	<0.001	
≥3.50	Ref	0.87 (0.83, 0.91)	<0.001	
Smoking				0.03
No	Ref	0.85 (0.81, 0.88)	<0.001	
Yes	Ref	0.90 (0.87, 0.93)	<0.001	
Drinking				0.54
No	Ref	0.88 (0.83, 0.93)	<0.001	
Yes	Ref	0.86 (0.84, 0.89)	<0.001	
Hypertension				0.21
No	Ref	0.87 (0.81, 0.94)	<0.001	
Yes	Ref	0.93 (0.88, 0.98)	0.01	
DM				0.02
No	Ref	0.85 (0.82, 0.88)	<0.001	
Yes	Ref	0.92 (0.87, 0.98)	0.01	

OA, osteoarthritis; eGDR, estimated glucose disposal rate; BMI, body mass index; PIR, poverty income ratio; DM, diabetes mellitus.

## 4 Discussion

This cross-sectional investigation leveraged the comprehensive demographic data available through NHANES to explore the association between eGDR and the prevalence of OA. A total of 9,051 individuals from the 2011 to 2018 NHANES surveys were included, of which 1,655 had been diagnosed with OA. Across different adjustment models, we noted that participants with reduced eGDR levels were more likely to have OA. This pattern held true in both continuous and categorical analytical approaches. Furthermore, after controlling for all potential confounders, a significant inverse relationship was identified between eGDR and the prevalence of OA, indicating a linear association. This significant negative relationship was also evident within various demographic subgroups. Additionally, our study revealed that the relationship between eGDR and OA prevalence was independent of gender, race/ethnicity, educational attainment, marital status, PIR, BMI, alcohol use, and hypertension, as demonstrated in the subgroup analyses(all interaction *P*-values >0.05). This indicates that the inverse relationship may extend to individuals with varying demographic characteristics. As far as we are aware, this is the initial investigation to uncover the connection between eGDR and OA within a substantial cross-sectional cohort of middle-aged and older adults in the United States.

Diabetes mellitus has become a worldwide public health emergency, experiencing marked shifts in its epidemiological profile in recent times. The global prevalence of diabetes is increasing, presenting a significant threat to personal well-being and healthcare infrastructure. Current estimates suggest that around 462 million adults across the globe are affected by diabetes, and projections indicate that this figure could rise to 700 million by the year 2045. Diabetes not only drastically affects the lives of those afflicted but also imposes a heavy financial strain on healthcare systems [21,22,23]. The associated medical costs and treatment expenses for complications of diabetes represent a significant economic burden. OA is considered a complex disease influenced by various factors. These include genetics, age, gender, obesity, joint injury, and metabolic disorders. Among these influencing factors, diabetes is regarded as a crucial causative factor, with a positive correlation observed between OA and type 2 diabetes [24,25]. However, this conclusion is subject to debate, as some studies argue that diabetes is not a risk factor for OA [26]. Within the context of this current research, following the exclusion of the impact of BMI, it was observed that eGDR continued to be inversely associated with the incidence of OA. Consequently, these findings endorse the concept that diabetes serves as a risk factor for the development of OA.

Diabetes mellitus is characterized by insufficient insulin secretion or an inadequate response of the body to insulin. Insulin, a hormone secreted by pancreatic β cells, primarily functions to regulate blood glucose levels by promoting the uptake of glucose by the liver, muscles, and fat tissues, thereby reducing blood glucose levels. IR refers to a decrease in the body’s response to insulin, where insulin cannot effectively promote the uptake and utilization of glucose by cells, leading to elevated blood glucose levels. IR is one of the main mechanisms of disease onset. Under conditions of IR, pancreatic β cells must secrete more insulin to maintain normal blood glucose levels. Prolonged overwork can lead to β-cell dysfunction, gradually reducing insulin secretion and eventually failing to meet the body’s needs, leading to elevated blood glucose levels and eventually developing into type 2 diabetes. There are various methods to assess IR, such as the euglycemic hyperinsulinemic clamp (the gold standard), the intravenous glucose tolerance test, and oral glucose or mixed meal tolerance tests. However, due to the time-consuming and cumbersome nature of these assessment methods, their clinical practicality and feasibility in large-scale epidemiological surveys are limited. The eGDR is a recently introduced index for IR that has gained attention for its simplicity and reliability. Many researchers advocate using eGDR as an indicator of IR. Recent studies have shown a strong correlation between eGDR and cardiovascular diseases [27,28], stroke [29], retinopathy [30], and kidney disease [31].

Recent studies indicate that the development of OA is linked to IR. Prior research has documented a connection between the TyG index and OA, suggesting that higher TyG index values are positively associated with the occurrence of OA [32]. For each unit rise in the TyG index, the likelihood of developing OA amplifies by 634% [32]. In certain instances, the TyG index seems to lack adequate sensitivity and specificity [33]. This research is the pioneering effort to examine the relationship between eGDR and OA. Previous studies have demonstrated that eGDR exhibits superior predictive power compared to the TyG index in the context of cardiovascular diseases. This superiority is likely due to the eGDR’s incorporation of both clinical and laboratory data in its computation, which offers a more nuanced evaluation of IR. Compared to traditional methods for assessing IR status, eGDR is non-invasive and less costly, making it more suitable for large-scale clinical applications. Moreover, eGDR has similar accuracy to the euglycemic hyperinsulinemic clamp in assessing IR status [34]. Our study found a negative correlation between eGDR and OA. Although the exact biological mechanisms by which IR leads to OA are not fully understood, several plausible explanations have been proposed. IR can promote the progression of OA through two important mechanisms: inflammation and metabolism. During the onset of OA, the production of TNF-α increases. Synovial fibroblasts in the joint generate IL-1β, IL-6, MMP1, and MMP13 in response to TNF-α. Hamada et al. [35] reported that insulin can inhibit the generation of inflammatory factors and proteases induced by TNF-α. In patients with IR, the signaling of insulin is impaired, weakening the anti-inflammatory effect of insulin. In patients with IR, due to long-term nutritional excess, including free fatty acids, nutrients can induce tissue inflammation [36]. Free fatty acids can induce TNF-α and TLR activation to promote the progression of OA [37,38,39,40]. Carnitine is a key molecule in mitochondrial energy generation [41]. Under continuous metabolic stress, carnitine deficiency affects the energy metabolism of articular cartilage [42,43]. Supplementation with carnitine can improve the clinical symptoms of patients with OA [44]. Many studies have found that anti-diabetic treatment can improve or delay the progression of OA. In future research, we can investigate whether treatments targeting IR can enhance the effectiveness of arthritis treatment in individuals with diabetes.

## 5 Limitations

Initially, given the observational design of the research, we are unable to infer causation, and a reciprocal causal link may even be present. Nonetheless, this appears to be improbable. Secondly, although our model made extensive adjustments for a multitude of potential confounders, the complete elimination of residual confounding remains a possibility, an inherent difficulty in the realm of observational studies. Furthermore, the outcome of interest in this study was dependent on self-reported data from participants, which were based on their physicians’ diagnoses. This reliance on self-reporting may introduce recall bias and lead to unavoidable misclassification. Despite this limitation, this approach is frequently used in cohort study methodologies.

## 6 Conclusion

An increased eGDR index is associated with a decreased risk of OA. The eGDR could potentially act as a significant biomarker for the identification of OA, providing a novel approach for evaluating and managing the disorder.

## Appendix

**Table A1:** Clinical characteristics grouped by eGDR quartiles

Variables	Overall	eGDR-Q1	eGDR-Q2	eGDR-Q3	eGDR-Q4	*P* value
Age, %	57.35 ± 0.22	60.19 ± 0.32	60.34 ± 0.32	56.93 ± 0.33	53.05 ± 0.33	<0.001***
**Race/ethnicity, %**						<0.001***
White	72.81(64.65, 80.97)	72.86(68.76, 76.95)	71.33(67.46, 75.21)	72.86(69.09, 76.63)	73.91(70.47, 77.34)	
Black	8.95( 7.67, 10.24)	12.46(9.64, 15.28)	9.71(7.87, 11.56)	8.24(6.52, 9.97)	6.14(4.90, 7.38)	
Mexican	6.26( 4.85, 7.67)	6.24(4.26, 8.21)	5.45(4.01, 6.89)	7.59(5.44, 9.74)	5.74(4.44, 7.05)	
Others	11.98(10.73, 13.23)	8.45( 6.93, 9.96)	13.50(11.24, 15.77)	11.31( 9.54, 13.07)	14.21(12.02, 16.40)	
**Education levels, %**						<0.001***
Less than high school	12.72(11.10, 14.35)	13.57(11.46, 15.67)	14.40(12.43, 16.38)	13.55(11.18, 15.93)	9.98(7.92, 12.05)	
High school or equivalent	22.18(19.77, 24.59)	27.21(24.35, 30.07)	22.83(19.95, 25.70)	22.69(20.17, 25.22)	17.16(14.94, 19.38)	
College or above	65.10(59.45, 70.75)	59.22(56.62, 61.83)	62.77(59.30, 66.25)	63.75(60.02, 67.48)	72.85(69.41, 76.29)	
**PIR, %**						<0.001***
≤1.30	16.43(14.40, 18.45)	20.21(17.85, 22.57)	17.16(14.76, 19.56)	17.09(14.17, 20.01)	12.22(10.08, 14.36)	
1.31–3.49	34.31(31.39, 37.24)	37.16(34.36, 39.96)	36.06(32.86, 39.26)	36.15(32.65, 39.66)	29.03(25.56, 32.49)	
≥3.50	49.26(44.02, 54.50)	42.63(39.09, 46.18)	46.78(42.62, 50.94)	46.76(42.63, 50.88)	58.75(54.21, 63.29)	
BMI, kg/m^2^	29.50 ± 0.12	36.05 ± 0.23	29.07 ± 0.15	29.28 ± 0.17	24.74 ± 0.10	<0.001***
Hemoglobin, g/dl	14.19 ± 0.03	14.30 ± 0.05	14.19 ± 0.05	14.24 ± 0.04	14.04 ± 0.04	<0.001***
waist circumference, cm	101.97 ± 0.28	119.27 ± 0.37	101.53 ± 0.33	101.73 ± 0.37	88.58 ± 0.21	<0.001***
HbA1c, %	5.81 ± 0.02	6.58 ± 0.04	5.75 ± 0.03	5.66 ± 0.02	5.39 ± 0.01	<0.001***
TC, mmol/L	5.15 ± 0.02	4.92 ± 0.04	5.15 ± 0.04	5.21 ± 0.03	5.27 ± 0.03	<0.001***
eGFR, mL/min/1.73 m^2^	85.77 ± 0.35	82.25 ± 0.61	82.84 ± 0.59	86.28 ± 0.48	90.48 ± 0.62	<0.001***
**Smoking, %**						<0.001***
No	54.22(50.31, 58.14)	47.73(44.43, 51.03)	51.86(48.63, 55.08)	52.45(49.07, 55.83)	62.89(60.23, 65.54)	
Yes	45.78(41.53, 50.02)	52.27(48.97, 55.57)	48.14(44.92, 51.37)	47.55(44.17, 50.93)	37.11(34.46, 39.77)	
**Drinking, %**						<0.001***
No	23.36(20.82, 25.90)	27.25(24.79, 29.71)	25.90(22.94, 28.85)	23.18(20.81, 25.55)	18.37(15.68, 21.06)	
Yes	76.64(70.83, 82.45)	72.75(70.29, 75.21)	74.10(71.15, 77.06)	76.82(74.45, 79.19)	81.63(78.94, 84.32)	
**DM, %**						<0.001***
No	80.06(73.81, 86.32)	50.32(47.76, 52.89)	81.20(78.66, 83.74)	86.72(85.00, 88.45)	97.35(96.56, 98.13)	
Yes	19.94(18.24, 21.63)	49.68(47.11, 52.24)	18.80(16.26, 21.34)	13.28(11.55, 15.00)	2.65( 1.87, 3.44)	
**OA**						<0.001***
No	78.79(72.97, 84.60)	69.41(66.30, 72.53)	74.74(72.34, 77.15)	81.74(79.36, 84.13)	86.98(84.79, 89.17)	
Yes	21.21(19.10, 23.33)	30.59(27.47, 33.70)	25.26(22.85, 27.66)	18.26(15.87, 20.64)	13.02(10.83, 15.21)	

Continuous data were presented as the mean±SEM, category data were presented as the proportion and 95% confidence interval. SEM, Standard Error of the Mean; eGDR, estimated glucose disposal rate; PIR, poverty income ratio; BMI, body mass index; HbA1c, glycosylated hemoglobin; TC, total cholesterol; eGFR, estimated glomerular filtration rate; DM, diabetes mellitus; OA, Osteoarthritis; *** *P* value < 0.001, ** *P* value < 0.01, * *P* value < 0.05.
